# Bifurcation PCI: keep it safe and simple!

**DOI:** 10.1007/s12471-017-0972-5

**Published:** 2017-02-27

**Authors:** P. Stella, J. Wykrzykowska

**Affiliations:** 1 Academic Medical Center Utrecht, Utrecht, The Netherlands; 2 Academic Medical Center Amsterdam, Amsterdam, The Netherlands

To the Editor,

We would like to compliment Leus and colleagues on their well-written retrospective analysis of their own PCI data from Catharina Hospital, Eindhoven, in 2013, which was published in a recent issue of this Journal [1].

However, we also would like to advise some caution in taking the discussion points and conclusions at face value as in the manuscript.

1. First of all, it should be very clear that this single-centre data retrospective analysis cannot be compared with multicentre RCTs such as Nordic, BBC, Cactus etc. By the very nature of this type of analysis, a natural selection bias cannot be excluded.
2. The authors mention that both groups are comparable in terms of baseline characteristics; however, looking at the angiographic baseline characteristics this is not the case: true bifurcations were present in 54.3% vs 100% of the patients treated with the one or two-stent approach, respectively. This provokes the discussion on selection bias since true side branch disease (>50% significantly diseased, extending more than 5 mm into the vessel) will potentially lead to the use of a two-stent technique.
3. The definition of a true bifurcation is not just the Medina classification!
4. Clinical restenosis rates are always lower than in aforementioned RCTs since angiographic follow-up always leads to a higher re-intervention rate. However, the MACE at one year is not so low (11.9 vs 12.2%), with a relatively high number of deaths and target vessel revascularisation. This underscores the fact that bifurcation PCI is ‘a different animal’ to non-bifurcation PCI.

To conclude we would like to state that this retrospective analysis confirms what has been shown in RCT: in the vast majority of bifurcation PCIs, a KISS (keep it safe and simple) approach is best (provisional approach). However, if a bifurcation becomes complex, complex techniques are needed and this paper nicely shows that even in these – selected – cases the overall outcomes vs simple one-stent bifurcation are comparable. If one chooses a complex technique, it is important is that one should try to master all its tips and tricks.
